# Editorial: Speech, language, and literacy development in individuals with Down syndrome

**DOI:** 10.3389/fpsyg.2023.1346494

**Published:** 2024-01-04

**Authors:** Sue Buckley, Kelly Burgoyne, Susan Loveall

**Affiliations:** ^1^Down Syndrome Education International, Kirkby Lonsdale, United Kingdom; ^2^Manchester Institute of Education, University of Manchester, Manchester, United Kingdom; ^3^Department of Special Education and Communication Disorders, University of Nebraska-Lincoln, Lincoln, NE, United States

**Keywords:** Down syndrome, speech, language, literacy, interventions

The ease and speed with which many children develop spoken language belies the complex processes that underpin it. Further, speech and language are fundamentally important for learning, thinking, reasoning, and remembering, as well as for communicating and fully participating in the social world. Mastering a written language is equally important and inextricably linked to spoken language, with each influencing progress in the other (Ellis and Large, 1988). Speech and language difficulties are common, and often persistent and severe, for individuals with Down syndrome, potentially impacting all aspects of cognitive and social development. This Research Topic contributes to our understanding of speech, language, and literacy development in individuals with Down syndrome and of effective interventions for this population.

Speech delays and difficulties are well-documented in individuals with Down syndrome, but there has been a lack of intervention work in this area. This may be linked to the field's limited understanding of underlying speech processes and their associated challenges. This is addressed in Madhaven et al. who propose a framework incorporating the biophysiological and environmental constraints to speech development and the interaction between them. This framework has implications for planning interventions for speech, language, and literacy. For example, they highlight the importance of somatosensory feedback children receive as they begin to produce sounds, which may be reduced in infants with Down syndrome.

As children with Down syndrome develop spoken language, many also develop dysfluencies, including stuttering. Although this is well-known in the field, there has been limited published research on this topic. In a review of the current literature, Hokstad and Næss identify that stuttering affects about 1 in 5 school age and adult individuals with Down syndrome and twice as many males as females but is less commonly diagnosed in pre-schoolers with Down syndrome relative to typically developing peers. They also note limitations of existing research and call for longitudinal studies to examine changes over time.

Turning to papers on language development, Mattie and Fanta explore the role of joint engagement in the vocabulary development of infants from 12 to 30 months. They report a perhaps unexpected finding that infants with more advanced joint engagement skills had fewer spoken words when measured concurrently and 9 months later. However, those with more advanced joint engagement skills at the later time point had higher receptive and expressive language scores. The authors suggest that at the earlier point in development, children may be using more advanced joint engagement behaviors to compensate for delayed spoken language and call for further research to unravel what is happening.

Two papers addressing early language development highlight the important role of shared book reading. Dulin et al. explore the home literacy environment (HLE) with a group of 11–14-month-olds using both a questionnaire and by recording and coding a parent-child shared book reading activity in the home. Richness of the HLE and quality of shared book reading activity, along with richness of the home language environment more broadly and the child's engagement in shared book reading, predicted receptive vocabulary 6 months later. Jeremic et al. review the literature on shared book reading as a language intervention for children with Down syndrome from birth to 6 years. They conclude that, despite limitations of existing studies, shared book reading can enhance children's language and communication. They identify that parents adapt their language for their child and that shared book reading provides opportunities for developing language. However, they also point out that it is possible that parents who are better at engaging their child in book reading may also provide higher quality language learning environments for their children throughout the day. This possibility should be considered in future longitudinal studies.

Romski et al. compare two interventions to teach first words to 24–29-month-olds over 12 weeks. Parents were coached in interventions embedded in natural play, in the clinic, and at home. One intervention focuses on teaching in a speech only format; the other includes the use of a speech generating device (SGD). The SGD group had more spoken words and intelligible utterances at the end of the intervention. The reasons for this warrant further study but may be the result of the SGD intervention providing more spoken repetitions of the target words.

Two studies look at later language development. Witecy et al. report on grammatical development from 4 to 17 years of age. Mastering verb agreement seems to be a prerequisite for mastering Wh- questions in German. They also report slower grammar learning from 10 years, suggesting there may be a critical period in which to develop grammar before this age. However, there are several possible explanations to be explored before reaching this conclusion. Children learn to talk so that they can communicate and may develop enough language to communicate effectively in their everyday world without needing to master grammar. More information about language learning environments and therapy experiences are also needed to inform understanding. The paper from Neitzel examines the narrative abilities of 10–20-year-olds. Patterns of individual differences in narrative responses suggest that those with more delayed non-verbal cognition and language abilities had more limited narrative abilities. There is a need to explore the narrative and communication abilities of young people in their everyday lives as well as experimental situations as these may differ.

Costanzo et al. report an intervention where Italian children and adults (5–29 years) were taught to communicate using an app which recognizes unclear speech and translates it into clear words. They report high levels of user satisfaction and some improvements in language abilities. A revolution in the way that technology may support this population may be seen in the near future, and this and the Romski et al. papers in this edition provide different examples of this.

Education and therapy services play a critical role in the progress and support of children and families, but the recent COVID pandemic brought these services to a halt. Pagnamenta et al. report the impact of this on families with children from 2 to 20 years. Parents reported that it was a stressful time, with some reporting a decline in their children's language and communication. What is learned from studies of the impact of COVID can be used to inform responses in future pandemics, but importantly, has also led to the positive development of tele-practice delivery of services.

On the assessment side, the paper from Channell et al. evaluates the validity of the Social Skill Improvement System Rating Scales for 124 individuals with Down syndrome from 6 to 17 years and demonstrates its validity and relations with other commonly used measures of behavior, autism traits, and executive functioning. The paper also highlights the relation between better expressive language abilities and social participation, and conversely, the link between poor expressive language and increased behavior difficulties. This highlights the role of language in all aspects of development, including behavior regulation and effective communication.

Moving into literacy, papers on early and adult readers include a study from Arango et al. which reports on the reading abilities and component skills of 6–10-year-old Chilean children learning to read Spanish. Measures included vocabulary, phonological awareness (PA), letter knowledge, sight word reading and verbal reasoning. Most measures showed improvement with age, but there was evidence of greater difficulty with PA and letter knowledge. This finding is consistent with studies of readers in the English language. Vocabulary was a predictor of word reading and some PA measures, which highlights the links between spoken language and reading. Importantly though, the authors point out that longitudinal studies are needed to explore these relations further.

Frizelle et al. report on co-construction of a reading assessment with 46 self-advocates in Ireland, examining what is considered relevant reading for them and constructing an accessible assessment. This is a pioneering example of inclusive research, involving the stakeholders from the outset and identifying what is relevant in their lives.

Not only does this Research Topic highlight the most recent research in speech, language, and literacy focused on individuals with Down syndrome, it also sets the stage for more impactful future research. As we see in this Research Topic, the field is moving beyond describing the communication difficulties of individuals with Down syndrome and into more research focused on interventions, meaningful and accurate assessments that do not demonstrate floor effects, community participation, and increasing diversity and the use of technology.

## Author contributions

SB: Writing—original draft. KB: Writing—review & editing. SL: Writing—review & editing.
